# APOE ε4 specific imbalance of arachidonic acid and docosahexaenoic acid in serum phospholipids identifies individuals with preclinical Mild Cognitive Impairment/Alzheimer's Disease

**DOI:** 10.18632/aging.101203

**Published:** 2017-03-23

**Authors:** Laila Abdullah, James E Evans, Tanja Emmerich, Gogce Crynen, Ben Shackleton, Andrew P Keegan, Cheryl Luis, Leon Tai, Mary J LaDu, Michael Mullan, Fiona Crawford, Corbin Bachmeier

**Affiliations:** ^1^ Roskamp Institute, Sarasota, FL 34234, USA; ^2^ University of Illinois at Chicago, Chicago, IL 60607, USA

**Keywords:** lipidomics, Alzheimer's disease, phospholipid, arachidonic acid, docosahexaenoic acid

## Abstract

This study was designed to explore the influence of apolipoprotein E (APOE) on blood phospholipids (PL) in predicting preclinical Alzheimer's disease (AD). Lipidomic analyses were also performed on blood from an AD mouse model expressing human APOE isoforms (EFAD) and five AD mutations and from 195 cognitively normal participants, 23 of who converted to mild cognitive impairment (MCI)/AD within 3 years. APOE ε4-carriers converting to MCI/AD had high arachidonic acid (AA)/docosahexaenoic acid (DHA) ratios in PL compared to cognitively normal ε4 and non-ε4 carriers. Arachidonic acid and DHA containing PL species, ε4-status and Aβ42/Aβ40 ratios provided 91% accuracy in detecting MCI/AD. Fish oil/omega-3 fatty acid consumption was associated with lower AA/DHA ratios even among ε4 carriers. High plasma AA/DHA ratios were observed in E4FAD compared to EFAD mice with other isoforms. In particular, alterations in plasma AA and DHA containing PL species were also observed in the brains of E4FAD mice compared to E3FAD mice. Despite the small sample size and a short follow-up, these results suggest that blood PL could potentially serve as biomarkers of preclinical MCI/AD.

## INTRODUCTION

The apolipoprotein E (APOE) ε4 allele is a major genetic risk factor for late-onset Alzheimer's disease (AD) which affects nearly 5 million individuals over the age of 65. Possession of an ε4 allele increases the risk of developing AD by 2-3 fold and occurs in nearly 60% of AD cases [1]. Compared to non-ε4 individuals, ε4 carriers exhibit brain amyloid pathology as early as age 40 and experience faster cognitive decline [2]. While the effects of the ε4 allele on brain AD pathology are known, it is unclear how the systemic effects of the ε4 allele contribute to the risk of developing AD. The apoE protein plays a key role in the transport and metabolism of lipids [3]. Therefore, investigation into the association between the APOE genotypes and blood lipids will be instrumental in determining their contribution to AD pathogenesis and their potential as biomarkers of preclinical AD, which are desperately needed to assist with early intervention efforts in AD.

Polyunsaturated fatty acids (PUFA) are required for maintaining the structure, function and vascular integrity of the brain [4, 5]. It has been proposed that the diminished capacity of apoE4 to transport PUFA contributes to the pathogenesis of AD [6]. The brain can synthesize non-essential fatty acids, but essential PUFA (e.g., arachidonic acid [AA] and docosahexaenoic acid [DHA]) are largely acquired from the peripheral circulation [7]. Arachidonic acid metabolism produces pro-inflammatory lipid metabolites, such as prostaglandins and leukotrienes, whereas metabolism of DHA generates anti-inflammatory mediators, such as resolvins [8, 9]. Hence, an increase in AA to DHA ratios could promote inflammation, further contributing to AD pathogenesis [9].

In blood, apoE transports lipids to various organs, including the brain [10]. Animal studies have shown that mice with human APOE4 display impaired transport of DHA to the brain compared to mice with human APOE2 [6]. Only a small percentage of PUFA exist as free fatty acids in blood and the unesterified forms of certain PUFA (i.e. DHA) can enter the brain [11]. Majority of PUFA are esterified to TG and PL that are complexed with apoE containing lipoproteins [12, 13]. Once lipoproteins reach the blood-brain-barrier (BBB), esterified lipids are processed by lipases present in the brain endothelial cells, resulting in the release of PUFA and their subsequent transport into the brain by simple diffusion or by specialized transporters [12, 14-16].

Major blood PL classes include phosphatidylcholine (PC), phosphatidylethanolamine (PE), phosphatidylinositol (PI) and lysophosphatidylcholine (LPC), where PC concentrations are highest relative to other PL classes [17]. Studies have shown that altered AA and DHA within blood PL are associated with AD diagnosis [18, 19]. However, it remains to be determined whether there are any ε4 genotype associated differences in AA and DHA containing PL between cognitively normal subjects and those with preclinical mild cognitive impairment (MCI)/AD. We hypothesized that an examination of AA and DHA content of PL classes alongside the APOE genotypes may predict preclinical MCI/AD. We performed lipidomic analyses of serum PL collected at baseline from a longitudinal cohort of cognitively normal individuals with a positive family history of late-onset AD or related dementia, of whom a subset converted to MCI or AD within 3 years. We also profiled plasma PL from mice with human APOE targeted replacement (APOE-TR) crossed with those harboring 5 familial AD mutations [20]. These studies suggest that the APOE genotypes together with AA and DHA within blood PL may be diagnostically important for detecting the preclinical stages of MCI/AD.

## RESULTS

### An increase in AA to DHA ratios preceded the diagnosis of MCI/AD

We examined serum PL profiles in a longitudinal cohort of 195 individuals, of whom 15 converted to MCI and 8 converted to AD while 172 subjects remained cognitive normal over 3 years. The APOE ε4 allele was present in 31% of subjects who remained cognitive normal, 40% among those who converted to MCI and 50% in those who converted to AD. There were no baseline differences between MCI and AD subjects for age, education, gender and ethnicity (p > 0.05). Cognitive scores at the annual visit conducted at 24 months post-enrollment showed that MCI and AD subjects had similar scores for MMSE, immediate and delayed Rivermead Behavioral and delayed recall (p > 0.05). Values of Aβ42/Aβ40 ratios in MCI subjects (0.11 ± 0.01 SE) were intermediate between controls (0.16 ± 0.02 SE) and AD subjects (0.05 ± 0.01 SE. Comparisons of the differences for general demographics between individuals who remained cognitive normal and those who converted to MCI or AD with or without ε4 (Table 1). Although within the normal limits, the MMSE scores were significantly lower among ε4 carriers converting to MCI/AD compared to other groups (Table 1). None of the ε4 and non-ε4 carriers converting to MCI/AD reported using fish-oil or omega-3 fatty acid supplements. Among ε4 non-carriers who remained normal, 14.5% consumed fish oil or another form of an omega-3 fatty acid supplement. About 9% of control ε4 carriers consumed fish oil/omega-3 fatty acid supplements (p < 0.05, Table 1).

**Table 1:** Baseline demographics of the study cohort stratified by the APOE ε4 carrier status

	APOE ε4- control (n =119)	APOE ε4- MCI/AD (n = 13)	APOE ε4+ control (n = 53)	APOE ε4+ MCI/AD (n = 10)
**N (%)**				
**Female**	60 (50)	6 (46)	29 (56)	2 (20)
**Caucasian**	117 (98)	13 (100)	51(96)	10 (100)
**Fish oil/Omega-3**	17 (14.3)	0 (0)	5 (9.4)	0 (0)
**Mean (± SE)**				
**Age**	76.8 (0.38)	79.1 (1.0)	76.23 (0.50)	78.0 (1.3)
**Education**	14.4 (0.27)	15.3 (0.90)	15.1 (0.33)	13.9 (0.96)
**MMSE**	28.8 (0.13)	29.0 (0.25)	29.2 (0.15)	27.8 (0.44)*

Note: * indicates p < 0.05 based on ANOVA.

**Figure 1:**
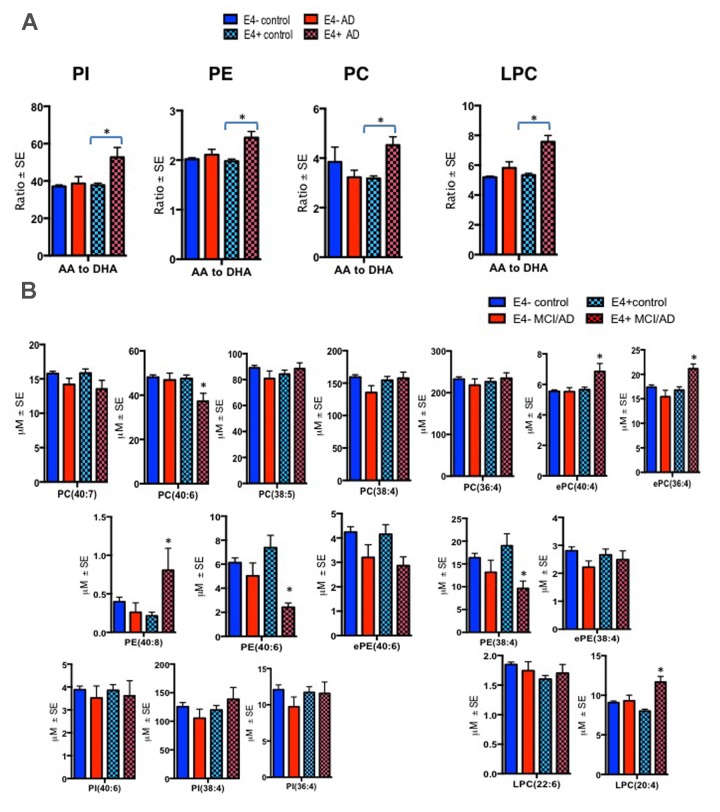
Ratios of AA to DHA and individual PL species stratified by diagnosis and the APOE ε4 carrier status Mean ± SE (ε4-non carriers = 119 control and 13 MCI/AD; ε4 carrier = 53 controls and MCI/AD = 10). (**A**) There was an interaction between MCI/AD diagnosis and ε4 allele for PC (F = 10.81, p = 0.001), PE (F = 4.95, p = 0.027), PI (F = 9.13, p = 0.003) and LPC (F = 15.05, p < 0.001). Subjects with the ε4 allele who later converted to MCI/AD had higher ratios of AA to DHA within PC, LPC, PI and PE relative to ε4 controls and ε4 non-carriers. (**B**) Individual AA and DHA species which significantly contributed to the imbalance in AA to DHA ratios among ε4 carries with MCI/AD compared to other groups include ePC(36:4), ePC(40:4), PC(40:6), PE(38:4), PE(40:6), PE(40:8) and LPC(20:4). *p < 0.05 for post-hoc analyses.

**Figure 2:**
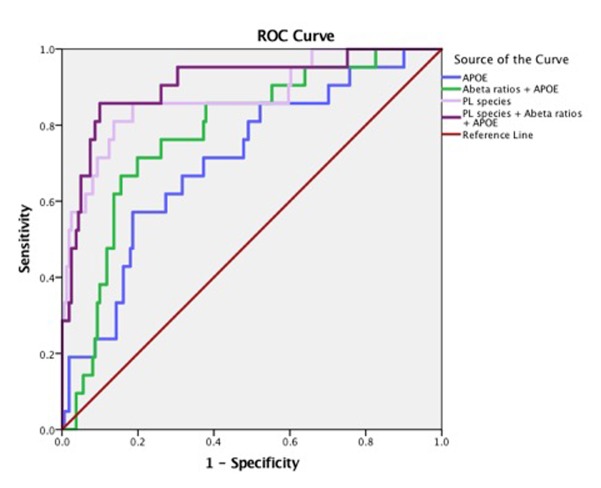
Arachidonic acid and DHA containing PL species along with ε4 carrier status and Aβ have high accuracy for predicting MCI/AD diagnosis Pilot ROC analyses were performed using the Cox-regression model consisting of a panel of PL that contained AA and DHA which included PE(36:4), PE(38:6), ePE(40:6), PE(40:6), LPC(20:4), LPC(22:6), ePC(36;4), ePC(40:4), ePC(40:6), PC(36:4), PC(38:4), PC(38:5), PC(40:4), PC(40:6), and PC(40:7). An AUC of 91% towards the diagnosis of MCI/AD was observed for this PL panel, ε4 and Aβ42/Aβ40 ratios. PL species alone provide an AUC of 88%. The APOE and ε4 together provided an AUC of 71%.

**Figure 3:**
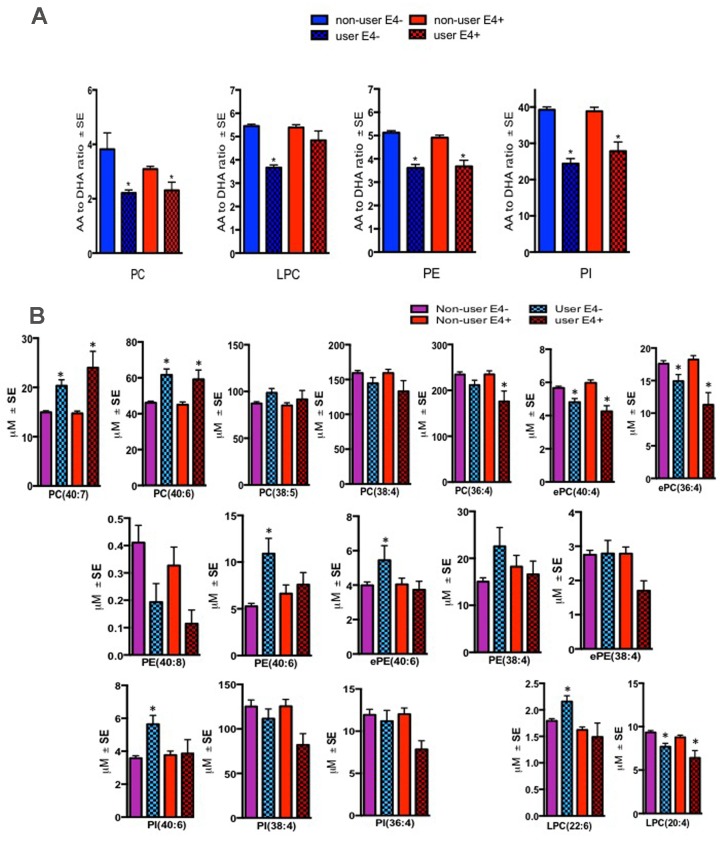
Effect of fish oil/omega-3 supplement use on the AA and DHA containing species within blood PL classes Mean ± SE(17 ε4- controls and 5 ε4 controls) for subjects who reported yes for using fish oil/omega-3 supplement. (**A**) Relative to non-users, ratios of AA to DHA were decreased in several PL classes in both ε4- and ε4+ controls who reported using fish-oil/omega-3 supplements*.* (**B**) Individual AA species (ePC(36:4), ePC(38:4), PC(36:4), LPC(20:4)) were generally decreased whereas DHA containing species (PC(40:6), PC(40:7), ePE(40:6), PE(40:6), and PI(40:6) were increased in several PL classes in both ε4- and ε4+ controls who reported using fish-oil/omega-3 supplements. While LPC(22:6) was increased in supplement users with ε4- genotype, levels of this lipid did not change in ε4+ individuals. *p < 0.05 for post-hoc analyses.

There were significant differences in the ratios of AA to DHA within PL between MCI/AD converters and those who remained normal and a confounding effect of the ε4 carrier status was observed (p < 0.05, Figure 1A). Supplementary Table 1 shows AA and DHA containing PL species, which contributed to high AA/DHA ratios among ε4 carrier AD patients compared to other groups. Within each PL class, there was a significant interaction between the ε4 carrier status and MCI/AD diagnosis, where *post-hoc* comparisons showed a significantly higher AA to DHA ratios among ε4 carriers who converted to MCI/AD compared to ε4 carriers who remained normal (p < 0.05, Figure 1A). Individual PL species contributing to the imbalance in AA to DHA ratios are presented in Figure 1B. Among ε4 carriers, ratios of AA and DHA within each PL class were similar in MCI and AD subjects compared to controls (Supplemental Figure 1). The distribution of total and individual PL species associated with MCI/AD conversion is presented in the Supplementary Table 1. The degree of unsaturation was not differentially modulated with respect to the ε4 carrier status and MCI/AD diagnosis (Supplementary Figure 2). Figure 2 shows a pilot receiver operator curve (ROC) constructed using a regression model adjusted for confounding factors and consists of AA and DHA containing species, ε4 status and Aβ42/40 ratios. This model has highest accuracy for predicting preclinical AD with an AUC of 91% (95% CI (83-93%)), whereas PL species alone provide a lower accuracy with an AUC of 88% (95% CI (78% to 98%)), followed by Aβ42/Aβ40 ratios and ε4 which provide an AUC of 78% (95% CI [68-88%]) and ε4 alone provided the AUC of 71% (95% CI [59-83%]). A significant effect of fish oil/omega-3 fatty acid supplementation was observed on the ratios of AA to DHA. In most instances, the ratios were decreased in ε4 and non-ε4 subjects reporting yes to prior use of omega-3 or fish oil fatty acid supplementation compared to those reporting no to using these supplements (p < 0.05, Figure 3A). Figure 3B shows decreases in AA containing species and increases in DHA containing PL species in individuals reporting yes for omega-3/fish-oil supplement use, even among ε4 carriers. We also examined the effects of the study interventions on AA and DHA containing PL species and found that the naproxen intervention increased PC(36:4) and PC(38:5) and decreased ePE(40:6) compared to the placebo group (p < 0.05, Supplementary Figure 3).

### An increase in AA to DHA ratios was observed in several PL classes in mice harboring APOE4 relative to other APOE isoforms

**Figure 4:**
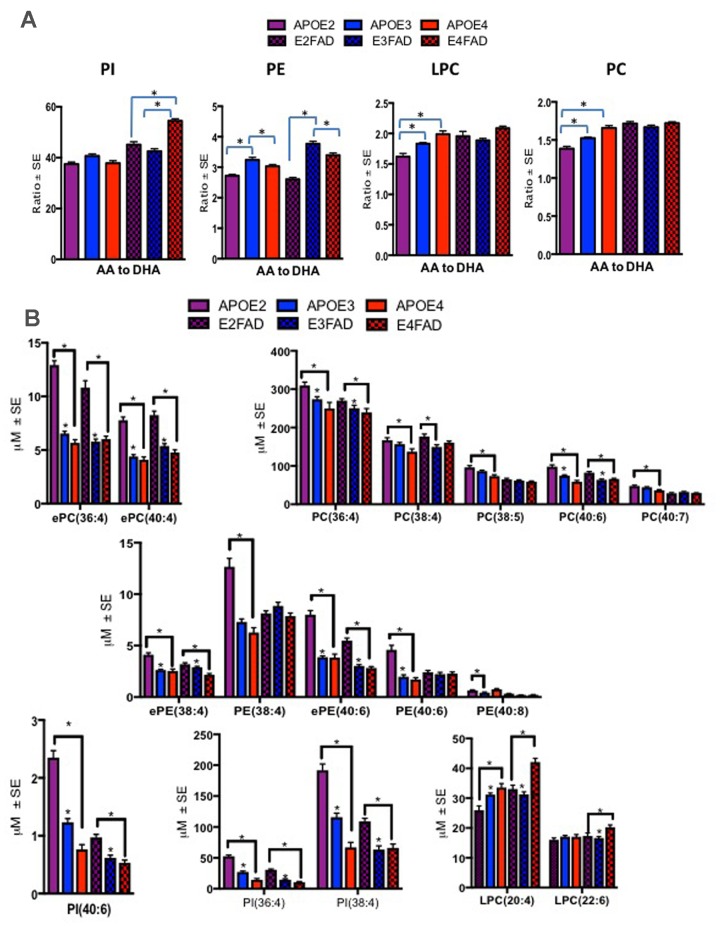
Ratios of AA to DHA and individual PL species stratified by APOE4-TR and E4FAD mice Mean ± SE (n = 6 per genotype). (**A**) There was a main effect of APOE genotypes on LPC (F = 10.53, p < 0.001) and PC (F = 33.73, p < 0.001). Relative to APOE2 and APOE3, ratio of AA to DHA containing species within PC and LPC were higher in APOE4 mice. There was also a main effect of the APOE genotypes in PE (F = 89.95, p < 0.001), ratios of AA to DHA were lower in APOE2 and APOE4 relative to APOE3 and ratios of AA to DHA were lowest in E2 FAD followed by E4FAD relative to E3FAD mice. There was also a main effect of the APOE genotypes for PI (F = 99.71, p < 0.001), where ratios were higher in E4 relative to E2 and E3FAD. (**B**) There were significant differences in various AA and DHA containing PL between the APOE genotypes for the APOE-TR and EFAD mice. While PL species, such as ePC(36:4), PC(38:4), ePE(38:4) and PI(36:4) were decreased in E4 relative to the other isoforms in APOE-TR and EFAD mice. LPC(20:4) and LPC(22:6) were increased in E4 mice relative to the other isoforms in APOE-TR and EFAD mice. *p < 0.05 for the post-hoc analyses.

These studies explored possible validation of human findings in a preclinical model of AD that contained both APOE isoforms and AD relevant genetic errors (EFAD mice). To date, this is the only model available that allows an examination of APOE together with AD pathological markers, amyloid and tau. Plasma from mice expressing human APOE isoforms (APOE-TR and EFAD) were subjected to lipidomic analyses to determine their translational value when compared to the APOE genotype related PL differences observed in human samples. The ratios of AA to DHA containing species were elevated in mice with E4 relative to other isoforms for both mouse models at 6 months of age. Most notably, ratios were significantly higher for E4 compared to other isoforms in LPC and PC in APOE-TR mice and for PI in E4FAD compared to E2FAD and E3FAD mice (p < 0.05, Figure 4). Figure 4B shows the profiles of PL species that contributed to the observed imbalance of AA to DHA ratios in the APOE4 and E4FAD compared to other isoforms in both APOE-TR and EFAD mice. Supplementary Table 2 provides a summary of mean levels of total and individual PL species across APOE-TR and EFAD mice that were differentially modulated in the human studies above. Most species were significantly higher in E2 relative to other isoforms in both models. However, LPC(20:4) which contains AA (LPC-AA) was higher in E4 compared to other isoforms in both mouse models, whereas LPC(22:6) that contains DHA (LPC-DHA) was higher only in E4FAD when compared to other EFAD mice. The degree of unsaturation of PL classes was differentially modulated in LPC with plasma polyunsaturated fatty acids (PUFA) were high only among E3FAD compared to E2FAD and E4FAD mice and monounsaturated fatty acid (MUFA) containing lipids were unaffected in EFAD mice (p < 0.05, Supplementary Figure 4). For the remainder of the PL classes, there was no differential modulation with respect to the degree of unsaturation since APOE2 and E2FAD were elevated compared to other genotypes, respectively (see Supplementary Figure 4). Supplementary Figure 5 shows a longitudinal profile of AA and DHA containing species, which contributed to AA/DHA ratio changes observed in E4FAD mice compared to E3FAD. Supplementary Figure 6 shows a reduction of certain AA and DHA containing PL species in the brains of E4FAD mice compared to E3FAD. However, LPC-AA and LPC-DHA were increased in plasma of E4FAD compared to E3FAD but decreased in the brains of E4FAD relative to E3FAD mice (Supplementary Figures 5 and 6).

## DISCUSSION

AA and DHA are critical for maintaining brain health and regulating inflammation [21, 22]. The adult brain relies heavily on the importation of AA and DHA from the periphery since their endogenous synthesis within the brain is low [23]. Until recently, it was thought that only unesterified DHA was able cross the BBB [11]. However, recent studies have demonstrated that esterified DHA can also cross the BBB and enter the brain via specialized lipid transporters [16]. Therefore, dysregulation of peripheral DHA homeostasis could result in decreased brain penetration, potentially contributing to AD pathogenesis [24]. Given the ε4 allele is the most potent genetic risk factor for AD and apoE facilitates lipid transport, we investigated the relationship between ε4 and blood PL on the risk of developing AD.

Our pilot ROC analyses show that when combined in a panel, AA and DHA containing species, Aβ42/Aβ40 ratios and ε4 genotype have an excellent accuracy in predicting MCI/AD. In our prior report, we showed that within 3 years, Aβ42/Aβ40 ratios predict conversion to MCI/AD with low accuracy unless combined with neuropsychological tests [25, 26]. Administration of neuropsychological tests requires considerable expertise, which is not routinely available in the community clinics. Thus, availability of a blood biomarker panel, such as the one proposed here, is appealing for the community settings. While generalization of our findings to a broader population is limited since our cohort was enriched for the presence of first-degree relatives with AD [25], such a strategy is useful for identifying subjects who are at a higher risk of developing AD and who may benefit the most from early interventions. As this study evaluated a short time period of about 3 years in a small cohort of 195 subjects, one can only infer a possible association of these PL species with an imminent risk of conversion to MCI/AD, which remains a limitation of this study. Since AD pathogenesis begins decades before the onset of clinical presentation [27], an evaluation of PL over a longer time period will determine their long-term predictive value in detecting AD.

We observed an increase in AA to DHA ratios within PC, LPC, PE and PI in ε4 carriers who converted to MCI/AD compared to cognitively normal ε4 subjects and non-ε4 carrier. The Framingham Heart Study reported that high blood DHA within PC was associated with reduced incidence of AD. However, this study did not comment on the AA content or the role of APOE genotypes [28]. Mapstone and colleagues reported that PC(38:6) and PC(40:6) (species containing DHA) were reduced in MCI/AD converters but they did not find any APOE genotype-dependent effects [29]. In another case-control study, PC(40:4), an AA containing species, was associated with a diagnosis of AD [30]. Findings reported by the AddNeuroMed consortium from a case-control study showed that these PC species together with the APOE genotype status can improve the accuracy for diagnosing AD [31]. Serum PE species containing AA and DHA were decreased in the pathologically confirmed AD cases compared to controls [32]. Mapstone et al., showed decreases in another form of PI in MCI/AD compared to controls [29]. To date, there have been no reports of an imbalance in AA to DHA within LPC in relation to APOE and MCI/AD diagnosis.

At 6 months of age, we found that APOE2 and E2FAD had much higher levels of PL compared to other genotypes in respective mouse models. The observed elevation would be consistent with the well-known role of APOE2 homozygous genotypes as a risk factor of type III hyperlipidemia [33]. At 6 months of age, an imbalance in AA and DHA was also observed in several PL classes in E4 mice compared to other isoforms in APOE-TR and EFAD mice. We also examined blood from EFAD mice at a range of ages covering 2.5 months to 18 months of age. At 6 months of age, extensive brain inflammation and amyloid pathology is observed in E4FAD mice relative to mice expressing other isoforms [34, 35]. This longitudinal profiling of E3FAD and E4FAD mice showed that several AA and DHA containing species were altered as early as 2.5 months of age, suggesting an association of these PL species with preclinical AD in this mouse model. An examination of E3FAD and E4FAD mice brain homogenates showed that several AA and DHA containing species that were elevated in blood (i.e. LPC(20:4), LPC(22:6), PC(40:6), and PC(38:4) of E4FAD mice compared to E3FAD mice were decreased in the brains of E4FAD mice compared to E3FAD mice. These studies suggest possible reduction in the transport of AA and DHA containing species by the apoE4 isoform. It is possible that lipid carrying capacity of APOE might be due to differences in apoE protein levels. For instance, previous studies have reported that levels of apoE protein are decreased in APOE4 compared to APOE3 mice (for both APOE-TR and EFAD mice) and in individuals who are carriers of the APOE ε4 allele compared to non-carriers [20, 36, 37]. In particular, studies have shown that plasma apoE levels are much lower among ε4 homozygous MCI subjects who convert to AD [38]. These studies support further examination of systemic AA and DHA imbalance in relation to the APOE genotypes and their contribution to AD pathology. As a number of advances have been made in application of lipidomics and metabolomics technologies [39, 40] and since bioactive lipid metabolites of AA and DHA have profound effects on modulating neuroinflammation, the use of these sophisticated technologies [41] to examine AA and DHA metabolites will be extremely valuable in understanding the role of AA and DHA containing PL in AD pathogenesis.

We were unable to examine the influence of statins and anti-hypertensive medications due to their confounding effects on MCI/AD [25], which required their incorporation as covariates in our analyses. We examined the effects of the study interventions on AA and DHA containing PL and found that only the naproxen intervention resulted in an increase in AA containing PC species and a decrease in an DHA containing PE species. This would be consistent with the inhibition of the cyclooxygenase pathways following the treatment with naproxen. However, owing to the fact that the study interventions did not modify the trajectory of AD onset following these treatments, it remains to be determined if there is any value of modulating AA containing PL species in preventing MCI/AD. However, possible confounding effects of the relationship between PL modulation by naproxen and AD prevention by other lipid modifying approaches cannot be excluded and require a larger sample size to examine this. We did however examine the effects of fish oil/omega-3 fatty acid supplementation in controls as none of the MCI/AD patients reported using these supplements. Fish oil or omega-3 fatty acid supplementation is a well-known modifier of DHA and has been put forth as a strategy to modulate AD progression [4]. Several randomized clinical trials have shown that DHA supplementation in elderly subjects improved verbal recognition memory and the immediate and delayed recall to the degree where it matched to the test norms of individuals 7 years younger [5]. This suggests that if DHA intervention was administered early, transition to pathologic aging could be reverted to normal aging. A prospective cohort study of 3000 elderly subjects in the Chicago Health and Aging study identified that unsaturated fat was negatively associated with the risk of developing AD [42]. Although these prospective studies show that the consumption of fish or fish-oil/omega-3 fatty acid supplements is protective against AD [4], they have not been consistently replicated in randomized clinical trials [43]. However, these studies did not account for APOE genotypes or baseline fish consumption or fish oil/omega-3 supplementation in their trial design. In our current study, an increase in AA containing and a decrease in DHA containing species corresponded with a decrease of AA to DHA ratios among subjects using these supplements compared to non-users for both ε4 carriers and non-carriers. These findings suggest that APOE genotype and fish oil/omega-3 fatty acid supplementation influence blood DHA levels and are consistent with a recent report showing that long-term DHA supplementation could affect AA and DHA levels even in APOE4 mice [44]. A recent animal study has shown that increasing brain DHA through diet can mitigate some aspects of inflammatory response to Aβ exposure [45]. Collectively, these studies suggest further exploration of the use of omega-3 fatty acid supplements strategy to intervene against the risk of developing AD among ε4 carriers.

Another factor driving the changes observed in peripheral DHA could be altered tissue distribution throughout the body, particularly in the brain. Recent rodent studies have shown that in healthy brains, more DHA reaches the brain when consumed as PL compared to TG [46]. Previous work has shown that LPC-DHA is a preferred carrier of DHA to the brain [47]. A more recent study showed that the major facilitator superfamily domain-containing protein 2 (mfsd2a) localized within the BBB uses electrochemical potential of sodium to preferentially transport LPC-DHA across the BBB [16]. In the absence of LPC-DHA, mfsd2a can transport LPC-AA across the BBB [16]. Another recent study showed that when peripherally administered, more LPC-DHA entered the brain the non-esterified DHA, partly because much more DHA is found esterified to LPC than non-esterified [11]. An increase in peripheral LPC-AA and LPC-DHA may be the result of impaired transport of this form of DHA into the brain. Recent studies have shown that DHA transport into the brain is impaired in mice with human E4 compared to E2 [6]. Our studies show that both LPC-AA and LPC-DHA were chronically elevated in E4FAD compared to E3FAD mice and LPC-AA was also elevated in ε4 carriers with preclinical MCI/AD compared to other groups. We propose that AA and DHA esterified to these PL should be examined to determine possible interactions with the APOE genotypes for their transit across the BBB. Future investigations into these aspects will likely clarify how the systemic effects of E4 and impaired lipid delivery to the brain contribute to AD pathogenesis.

In the present studies, we show an interaction between the ε4 status and high AA to DHA ratios with the risk of developing MCI/AD. We also demonstrate that combining the APOE genotypes, blood AA and DHA species and the Aβ42/Aβ40 ratio improves the accuracy for detecting preclinical MCI/AD. As such, this biomarker panel could be valuable for diagnosing preclinical MCI/AD. Such strategies for diagnosing AD at the pre-symptomatic stages are critically needed to advance early intervention efforts for preventing AD. Further examination of the interaction between APOE and PL for their collectively contributions to AD etiology is necessary in a larger cohort and over a longer follow-up period to fully ascertain the value of PL profiling in detecting preclinical AD.

## METHODS

### Subject selection

As part of an IRB approved ancillary study, samples were collected and banked from a subset of participants (n = 195) recruited from the Alzheimer's Disease Anti-inflammatory Prevention Trial (ADAPT), a randomized, placebo-controlled, multi-center primary prevention trial. Subjects were randomly assigned to one of three groups: celecoxib (200 mg b.i.d.), naproxen sodium (200 mg b.i.d.) or placebo with an allocation ratio of 1:1:1.5, respectively, which was maintained in this sub-cohort (43% on placebo, 28% on celecoxib and 29% on naproxen). There was no effect of the study intervention on the diagnosis of MCI or AD [48] so we used all three intervention arms for this study. Full details of the data collection, measurements, and study procedures are available elsewhere [49]. A separate consent was obtained from each subject who participated in this sub-study.

Study participants were selected based on the presence of a first-degree relative with AD-like dementia. Participant eligibility was determined using a study specific standard questionnaire, a physical exam and a brief neuropsychological battery [25]. Although the original study design did not intend to recruit subjects who were related to each other, an incidental recruitment of subjects who might be related to each other is possible. We expect that such enrichment would be randomly distributed between controls and cases since the study was a randomized controlled trial. Blood draws were conducted at the semi-annual visits and the cognitive status was captured on an annual cognitive assessment. Data on medication use and supplement (i.e. fish oil) use was collected based on self-report. The cognitive measures completed at baseline and annual follow-up included the Modified Mini-Mental State Examination [50], the Hopkins Verbal Learning Test-Revised [51] and the Wechsler Adult Intelligence Scale-Revised (WAIS-R; [52]), see details elsewhere [26]. Collateral respondents completed the Dementia Severity Rating Scale [53].

Individuals scoring below the normal cut-off underwent a dementia work-up including the physical and neurological examinations, laboratory studies and neuroimaging as applicable [26]. A comprehensive neuropsychological assessment was administered as part of the dementia work-up. This battery of tests included the expanded Consortium to Establish a Registry for Alzheimer's Disease battery [54]; Logical Memory I and II of the Wechsler Memory Scale–Revised (Wechsler), and Benton Visual Retention Test (Benton), see additional details elsewhere [26]. A consensus team provided a diagnosis of AD in accordance with the NINCDS-ADRDA [55]. Amnestic MCI had impairment of at least one cognitive domain, including memory, executive function, attention, language, and visuospatial skill and had preserved independent living activities, consistent with the Petersen criteria [56]. Efforts were made to rule other causes for cognitive impairment such as vascular, traumatic and other medical causes of cognitive decline as per Albert and colleagues [57]. Amnestic MCI patients are in a transitional stage between normal aging and AD, with 85% of MCI subjects converting to AD within 7 years [58]. An imaging study showed that patterns of brain atrophy in amnestic MCI patients is typical of that observed in AD patients [59]. Therefore, owing to a small sample size of subjects who converted to either MCI or AD, we combined these diagnostic categories to facilitate adequate sampling for estimating disease progression.

### Animals

Each mouse was housed in a controlled environment (12-hr light/dark cycle) and maintained on a standard diet. All animal experiments were approved by the Roskamp Institute Institutional Animal Care and Use Committee and conducted in accordance with the Office of Laboratory Animal Welfare and the Association for the Assessment Accreditation and Laboratory Animal Care guidelines. Transgenic mice expressing 5 familial AD mutations (5 x FAD) were crossed with APOE-TR mice (5 x FAD+/− human APOE+/+ (E2FAD, E3FAD, E4FAD)) as previously described [60]. Briefly, to generate EFAD mice, 5 x familial AD mice were bred with homozygous APOE2, APOE3 and APOE4-TR mice (Taconic Laboratories). Male APOE-TR homozygous mice (background C57BL6) were bred with female 5 x FAD heterozygous (background C57B//B6x SJL). The resulting female with mouse-APOE/APOE-TR/FXFAD were then backcrossed with male APOE-TR mice to produce APOE-TR homozygous/5xFAD heterozygous mice. For this study, 6-month old male EFAD and 4-5 month old male APOE-TR mice were utilized (n = 6 per group). Biochemical analyses show that male FAD mice accumulate significant extracellular Aβ in the subiculum and the frontal cortex from 2-6 months, with E4FAD > E3FAD = E2FAD. At 6 months of age, compared to E3FAD, E4FAD mice exhibited neuroinflammation, which is characterized by a higher level of microgliosis and astrogliosis [34, 61]. A greater load of cerebral amyloid angiopathy and amyloid plaque in the cortex is evident in 7-month old female E4FAD relative to E2FAD and E3FAD male and female mice [62] and a greater cognitive impairment in the E4FAD mice than in E3FAD and E2FAD mice [63]. To determine PL changes with age in relation to APOE and AD pathology, we also examined plasma from 2.5 months and 18 months old (male/female) E3FAD and E4FAD mice (n = 5/6 mice per group for each age). Due to difficulties in breeding mice with the APOE2 genotypes, E2FAD mice were not available for these studies [64]. Cortical brain homogenates were prepared as previously described [20]. Briefly, frozen cortices from dissected brains were homogenized in 15 volumes (w/v) of TBS, then centrifuged (100,000 x g, 1hr at 4 °C), after which the pellet was washed in TBS, resuspended in again in 15 volumes of TBS buffer containing 1% TBSX and mixed gently by rotation at 4 °C for 30 min. Samples were centrifuged again (100,000 x g, 1hr at 4 °C) and TBSX soluble fraction was collected and frozen in −80°C for future studies [20]. Total protein content in the TBSX extractions was determined via colorimetric BCA assay per the manufacturer's instructions.

### Sample collection, preparation and measurements

In human subjects, non-fasting blood draws were conducted by trained phlebotomists. Serum from blood of consenting subjects was processed using standard laboratory procedures [65]. To obtain mouse plasma, mice were exsanguinated via cardiac puncture using an 18-gauge wide-bore needle to prevent hemolysis of red blood cells (RBC) during blood collection. Blood samples were collected into a 1.5 ml Eppendorf tube containing EDTA. Samples were immediately centrifuged at 3000 x g for 5 min and plasma was snap frozen in liquid nitrogen.

### Lipidomics

The Folch extraction of serum and plasma samples was performed as previously described [66-68] using the Folch method [69] following an addition of di-14:0 fatty acid (FA) containing PC and PE (Matreya, Pleasant Gap, PA), di-16:0 PI, and 14:0 LPC (Avanti, Albaster, AL) as internal standards. High-pressure liquid chromatography (HPLC) was achieved using a 1mm ID x 10 cm column containing Pinnacle II 3 μm silica particles (Restek, Bellefonte, PA, USA). A gradient was run from 10% solvent B (80% methanol, 10 mM formic acid, 5 mM ammonium hydroxide) in solvent A (chloroform-acetonitrile, 2:1) to 55% B in A for 12 min with a 5 min hold at the final conditions. The flow rate was 50 μl/min with the column temperature at 40^o^ C. Mass spectrometry was performed with a Thermo LTQ linear ion trap mass spectrometer (Thermo-Fisher). Alternant positive and negative ion spectra were acquired from m/z 75 to 2,000 with in-source collision induced dissociation (SCID) and relative energies of 85% (positive ion) and 40% (negative ion). All spectra were obtained with a 200 ms maximum ion time and by summing 5 microscans. Mass spectra were summed over the chromatographic peak for each PL class, converted to listings with a threshold of 0.01% base ion intensity, exported to Microsoft Excel and then analyzed using LipidomeDB online to identify and quantify (with reference to the added internal standards) each PL molecular species [70]. Within each class, molecular species identified were totaled to generate total PC, PE and PI values. Each PL class was then analyzed and individual species containing saturated fatty acid (SFA), monounsaturated fatty acid (MUFA) or polyunsaturated fatty acid (PUFA) were grouped separately to generate a composite variable for each category. For PC, ratios were calculated using docosahexaenoic acid (DHA)-containing species, PC38:6 (16:0/22:6), PC40:6 (18:0/22:6) and PC40:7 (18:1/22:6), to arachidonic acid (AA)-containing species, ePC36:4 (16/20:4), ePC40:4 (20:0/20:4) PC36:4 (16:0/20:4), PC38:4 (18:0/20:4), and PC38:5 (18:1/20:4). For PE, a ratio of DHA-containing species, PE40:6 (18:0/22:6) and ether PE (ePE40:6, (18:0/22:6), to AA-containing species, PE36:4 (16:0/20:4) ePE36:4 (16:0/20:4), PE38:4 (18:0/20:4) and ePE38:4 (18:0/20:4), was calculated. For PI, a ratio of DHA-containing PI40:6 (18:0/22:6) to AA-containing PI36:4 (16:0/20:4) and PI38:4 (18:0/20:4) species was examined.

### Statistical analyses

For human samples, baseline differences were compared across the study population using either the Student's t-test or the Chi-square statistics. Most PL classes were normally distributed but a few PE species were log transformed for normalization. Lipidomic data were analyzed using principal component analysis (PCA) followed by mixed linear model (MLM) as described previously [67] to examine the independent effects of the APOE genotypes and MCI/AD diagnosis and any potential interaction between them on PL profiles. Post-hoc correction for multiple hypothesis testing was performed using Benjamini–Hochberg procedure (B-H) on p-values obtained using the least significant differences. Cox regression modeling was used to test whether AA and DHA containing PL species, the ε4 allele and Aβ42/Aβ40 ratios alone or in combination can predict conversion to MCI/AD. For Cox regression, age, education, gender, creatinine and treatment with statins or anti-hypertensive medications were included as covariates to limit their confounding effects [25]. We performed pilot receiver operator curves (ROC) analyses to assess each model in predicting the diagnosis of MCI/AD. Mouse data were analyzed using MLM regression as detailed above. All statistical analyses were performed using SPSS version 23 (IBM, NY, US).

## SUPPLEMENTARY MATERIALS FIGURES AND TABLES



## Abbreviations

AD: Alzheimer's disease
MCI: mild cognitive impairment
PL: phospholipid
PC: phosphatidylcholine
PE: phosphatidylethanolamine
PI: phosphatidylinositol
LPC: lyso-phosphatidylcholine
ROC: receiver operator curves.
