# Supplementary material for: APOE ε4 specific imbalance of arachidonic acid and docosahexaenoic acid in serum phospholipids identifies individuals with preclinical Mild Cognitive Impairment/Alzheimer's Disease
Source: Aging (Albany NY). 2017 Mar 23;9(3):964–78. doi: 10.18632/aging.101203 (PMC5391242; doi:10.18632/aging.101203)
Supplement: Supplementary file 1 [file aging-09-964-s001.pdf]

SUPPLEMENTARY MATERIALS

Supplemental Figures.

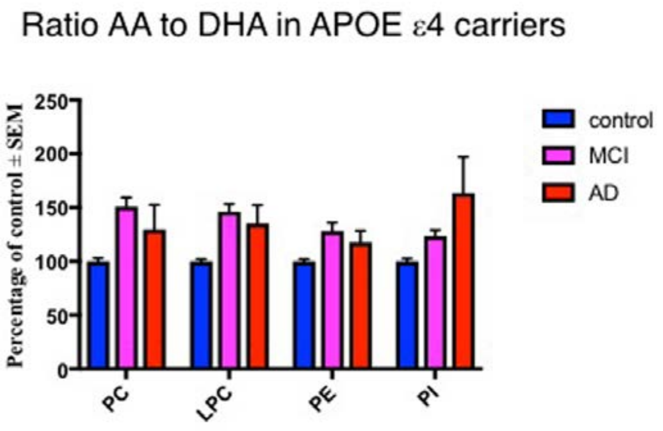

Supplemental Figure 1. Ratio of AA to DHA are similar in ε4 carriers with MCI and AD compared to ε4 controls. Mean ± SEM (ε4 carrier = 53 controls, MCI =6AD = 4). With PC, LPC, PE and PI, ratios of AA to DHA containing species were increased in both MCI subjects and AD patients.

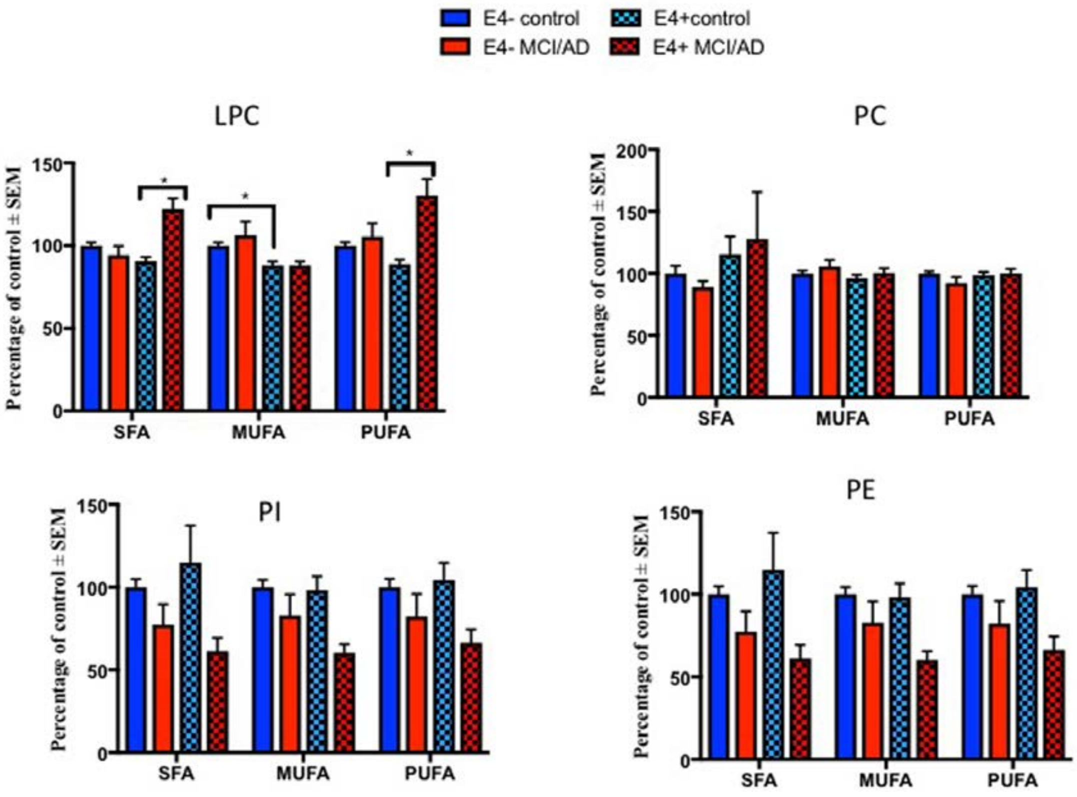

Supplemental Figure 2. Differential modulation of the degree of unsaturation of PL classes stratified by diagnosis and the APOE ε4 carrier status. Mean ± SEM (ε4-non-carriers = 119 control and 13 MCI/AD; ε4 carrier = 53 controls and MCI/AD = 10). Figure shows that there were no differences in the degree of unsaturation of PC, PE and PI across different diagnostic categories, even if stratified by the APOE ε4 carrier status. For LPC, all SFA, MUFA and PUFA elevated in ε4 carriers with MCI/AD compared to control subjects. *p < 0.05 for post-hoc analyses.

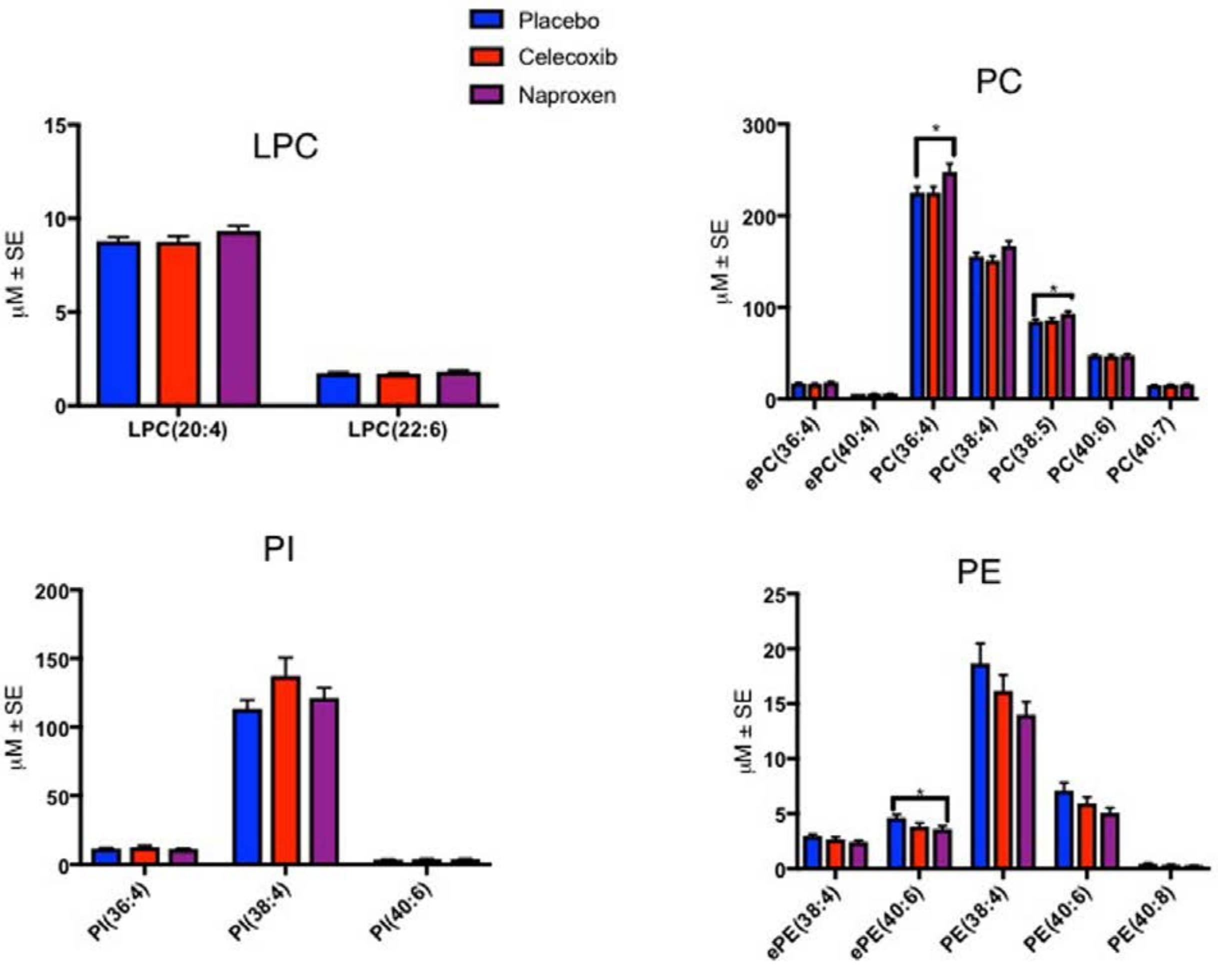

Supplemental Figure 3. Effect of NSAID intervention on AA and DHA containing PL species. Mean ± SEM (n = 198). There was no effect of celecoxib on any of the AA and DHA containing PL species when compared to the placebo group. Intervention with naproxen elevated several AA containing species (PC(36:4) and PC(38:5) and decreased DHA containing PE (ePE(40:6)) compared to placebo. *p < 0.05 for the post-hoc analyses.

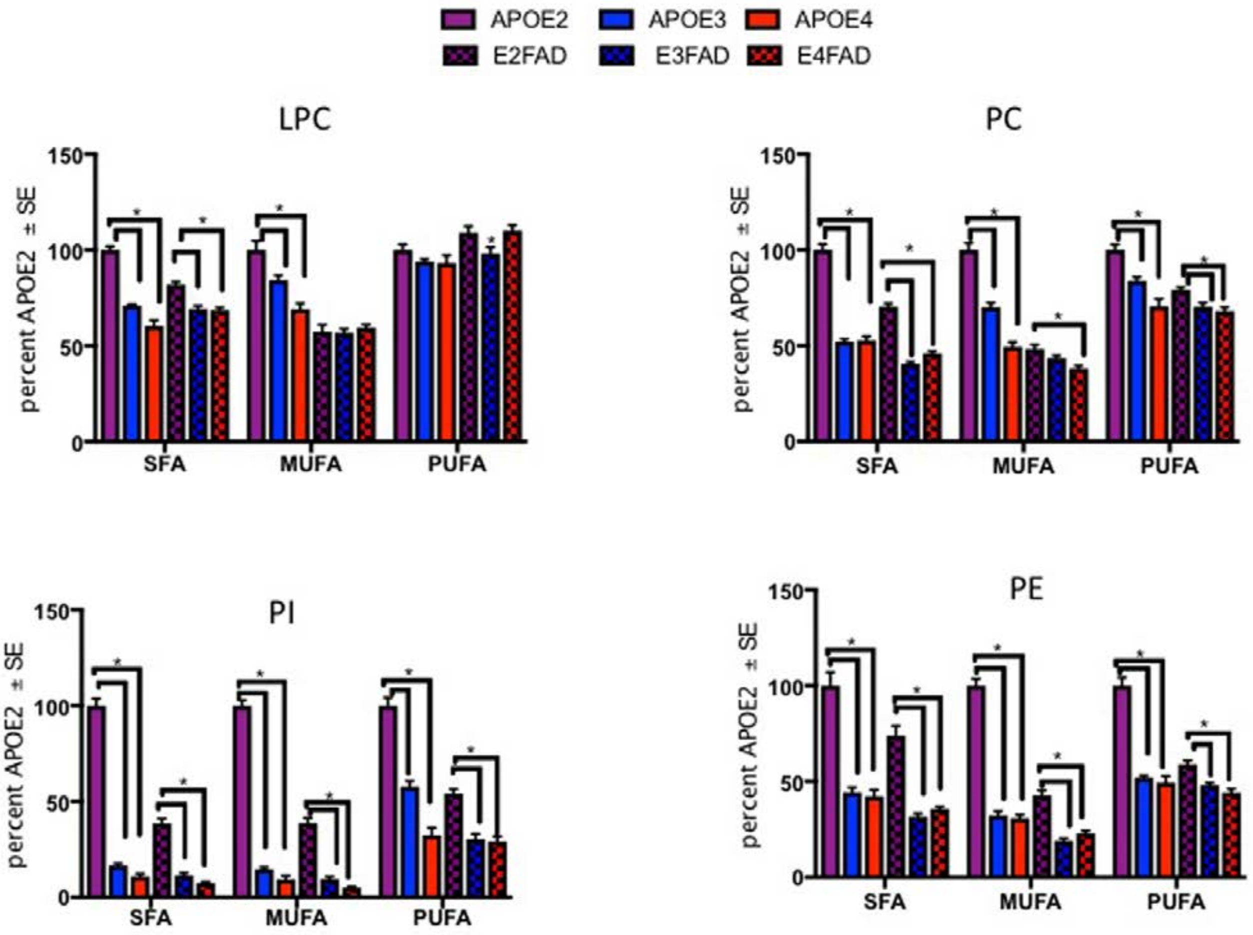

Supplemental Figure 4. Degree of unsaturation of PL classes stratified by the APOE genotypes in APOE-TR and EFAD mice. Mean ± SEM (n = 6 per genotype). For most PC, PE and PI classes, SFA, MUFA and PUFA were elevated in the E2 compared other isoforms in APOE-TR and EFAD mice. For LPC, E2 had elevated levels of SFA compared to other genotypes for both APOE-TR and EFAD mice. For MUFA within LPC, only APOE2 was higher than APOE3 and APOE4 isoforms. However, PUFA were elevated in E4FAD compared to E3FAD only. *$p < 0.05$ for post-hoc analyses.

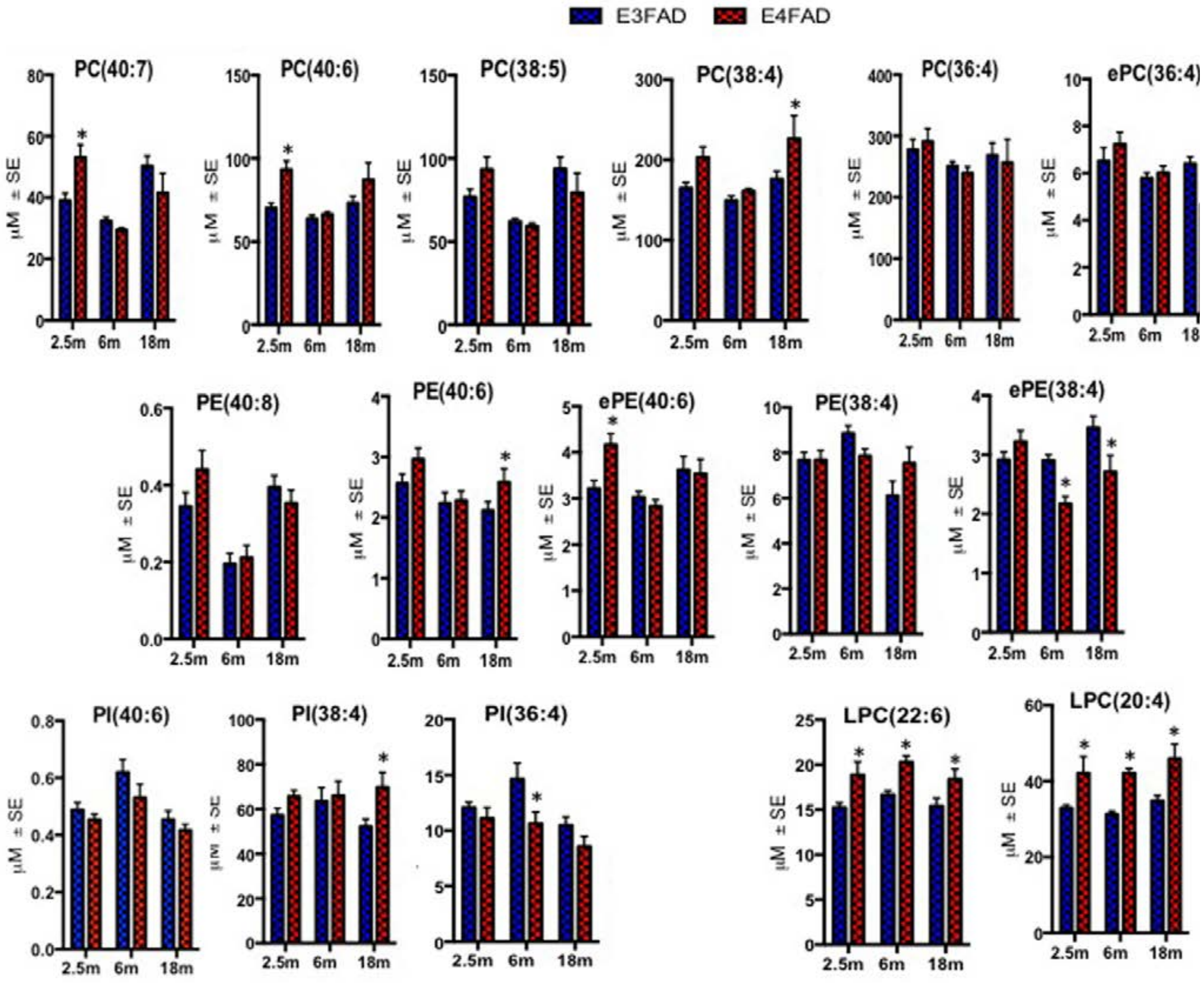

Supplemental Figure 5. Longitudinal plasma profiles of AA and DHA containing PL species in E3FAD and E4FAD mice. Mean ± SEM (n = 5/6 per genotype for each age group). There were significant differences between E3FAD and E4FAD with age for the levels AA containing PL species: ePC(36:4) PC(38:4), ePE(38:4), PI(36:4) and LPC(20:4) and for DHA containing species: PC(40:6), PC(40:7) ePE(40:6), PE(40:6) and LPC(22:6). *denotes $p < 0.05$ for the post-hoc analyses.

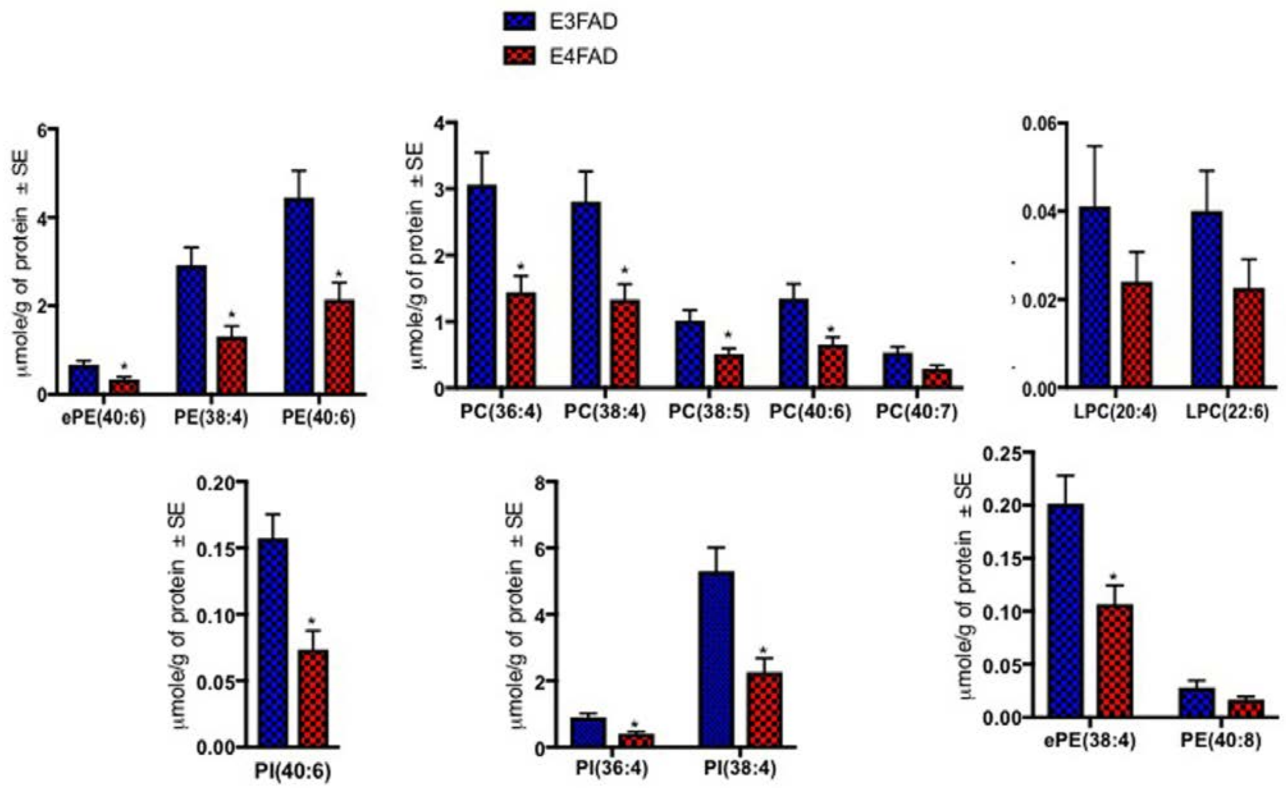

Supplemental Figure 6. Brain profiles of AA and DHA containing PL species in E3FAD and E4FAD mice. Mean ± SEM (n = 3/4 per genotype). Relative to E3FAD, E4FAD mice had lower AA containing PL species (ePC(36:4) PC(38:4), ePE(38:4), PI(36:4) and LPC(20:4) and for DHA containing species (PC(40:6), PC(40:7) ePE(40:6), PE(40:6) and LPC(22:6). *p < 0.05.

Supplementary table 1A. Total PL levels in control and MCI/AD subjects stratified by the APOE ε4 allele.

	ε4- control	ε4- MCI/AD	ε4+ control	ε4+ MCI/AD
Phospholipids	μM ± SE			
Total PC	2246 (38.9)	2203 (99.2)	2248 (54.6)	2279 (74.1)
Total LPC	187 (3.3)	184 (11.6)	168 (4.4)	234 (13.4)*
Total PE	163 (7.9)	138 (23.8)	171 (16.4)	109 (12.8)
Total PI	233 (11.2)	225 (13.3)	206 (27.8)	265 (37.4)

Note: * indicates p < 0.05 for interactive or confounding effect of APOE and diagnosis.

Supplementary table 1B. Phospholipid species in control and MCI/AD stratified by the APOE ε4 allele.

	ε4- control	ε4- MCI/AD	ε4+ control	ε4+ MCI/AD
Phospholipids	μM ± SE			
ePC(32:2)	3.1 (0.07)	2.9 (0.20)	2.8 (0.11)	3.3 (0.23)*
ePC(34:0)	2.4 (0.10)	2.5 (0.27)	2.1 (0.13)	3.0 (0.27)*
ePC(34:1)	8.0 (0.26)	7.2 (0.59)	7.6 (0.28)	9.2 (0.44)*
ePC(34:2)	9.7 (0.26)	9.1 (0.80)	9.3 (0.36)	11.5 (0.74)*
ePC(36:2)	13.8 (0.35)	12.0 (0.95)	14.2 (0.52)	15.9 (1.11)*
ePC(36:3)	8.3 (0.22)	7.2 (0.66)	7.8 (0.33)	10.6 (0.68)*
ePC(36:4)	17.4 (0.44)	15.4 (1.29)	16.8 (0.67)	21.1 (1.00)*
ePC(38:2)	1.9 (0.04)	1.8 (0.07)	1.8 (0.05)	2.3 (0.12)*
ePC(38:6)	13.3(0.29)	11.4 (0.83)	12.6 (0.41)	13.7 (0.66)
ePC(40:4)	5.5 (0.10)	5.5 (0.27)	5.6 (0.17)	6.9 (0.51)*
PC(38:6)	105.1 (2.32)	101.8 (5.72)	103.1 (3.34)	80.0 (7.36)*
PC(40:4)	6.6 (0.13)	6.6 (0.40)	6.4 (0.20)	7.1 (0.37)
PC(40:6)	48.1 (1.03)	46.9 (3.09)	47.5 (1.62)	37.3 (3.58)*
PC(40:7)	15.8 (0.33)	14.2 (0.89)*	15.8 (0.60)	13.5 (1.25)*
PC(42:11)	3.6 (0.08)	3.1 (0.28)	3.6 (0.14)	4.7 (0.32)*
eLPC(16:0)	1.4 (0.03)	1.23 (0.06)	1.3 (0.03)	1.8 (0.09)*
eLPC(18:0)	1.7 (0.04)	1.5 (0.10)	1.5 (0.06)	2.1 (0.15)*
LPC(16:0)	75.9 (1.51)	73.0 (4.40)	68.7 (2.00)	92.4 (4.98)*
LPC(16:1)	1.6 (0.06)	1.6 (0.19)	1.4 (0.07)	2.0 (0.17)*
LPC(18:0)	39.8 (0.74)	36.3 (2.1)	36.1 (0.98)	48.8 (2.49)*
LPC(18:1)	22.7 (0.43)	24.3 (1.90)	20.1 (0.51)	29.9 (2.01)*
LPC(18:2)	27.3 (0.58)	28.9 (2.44)	24.2 (0.72)	36.3 (3.11)*
LPC(20:5)	4.2 (0.13)	4.8 (0.35)	3.9 (0.18)	5.6 (0.47)*
LPC(20:4)	9.1 (0.19)	9.3 (0.71)	8.0 (0.23)	11.7 (0.71)*
LPC(22:6)	1.8 (0.04)	1.7 (0.15)	1.6 (0.06)	1.7 (0.15)
ePE(36:5)	10.0 (0.51)	6.8 (1.10)*	9.9 (0.94)	6.4 (0.81)*
ePE(40:6)	4.2 (0.22)	3.2 (0.52)*	4.2 (0.39)	2.9 (0.36)*
PE(36:2)	11.7 (0.61)	13.3 (3.44)	12.7 (1.28)	8.0 (0.92)*
PE(36:4)	8.2 (0.44)	7.4 (1.28)	8.7 (1.05)	5.5 (0.64)*
PE(38:5)	8.0 (0.46)	7.4 (1.61)	8.9 (1.05)	5.1 (0.68)*
PE(38:6)	10.2 (0.62)	8.3 (1.51)*	12.0 (1.54)	4.2 (0.50)*
PE(40:6)	6.1 (0.38)	5.0 (1.07)*	7.4 (1.02)	2.4 (0.35)*
PI(36:0)	0.51 (0.03)	0.44 (0.05)	0.50 (0.05)	0.6 (0.07)*
PI(36:1)	9.3 (0.33)	9.7 (1.06)	9.1 (0.51)	12.0 (1.61)*
PI(36:2)	30.5 (1.05)	30.1 (3.15)	31.2 (1.81)	41.4 (5.88)*
PI(36:3)	9.0 (0.33)	8.5 (0.94)	9.1 (0.52)	11.3 (1.38)
PI(38:5)	13.3 (0.66)	10.4 (1.45)*	12.4 (0.71)	15.2 (1.99)

Note: These individual molecular species were part of the PCA significantly associated with MCI/AD diagnosis. *indicates p < 0.05 for interactive or confounding between APOE and diagnosis on PL levels. There was a main diagnosis effect for PC(40:7), ePE36:5, ePE40:7, PE38:6 and PE(40:6).

Supplementary table 2A. Total PL stratified by APOE genotypes in APOE-TR and EFAD mice.

	APOE2	APOE3	APOE4	E2FAD	E3FAD	E4FAD
	µM ± SE					
Total PC	3098 (57.4)	2403 (48.2)*	2016 (110.2)*	2515 (46.4)	2122 (63.2)	1976 (63.1)*
Total LPC	557 (12.9)	440 (4.0)*	392 (17.6)*	482 (10.6)	422 (11.0)	440 (9.3)*
Total PE	184 (7.6)	86 (2.3)*	79 (5.5)*	110 (3.5)	80 (1.8)*	72 (3.3)*
Total PI	384 (15.0)	195 (9.9)*	110 (13.6)*	202 (7.2)	104 (9.6)	96 (9.2)

Note: * indicates $p < 0.05$ for higher E2 vs. E3 and E4 for APOE-TR and for higher E2FAD vs. E3 and E4FAD. ** indicates $p < 0.05$ for higher E4 vs. E2 and E3 for APOE-TR and for E4FAD vs. E2FAD and E3FAD.

Supplementary Table 2B. Phospholipid species stratified by APOE genotypes in APOE-TR and EFAD mice.

	APOE2	APOE3	APOE4	E2FAD	E3FAD	E4FAD
	µM ± SE					
ePC(34:0)	1.2 (0.12)*	0.4 (0.07)	0.7 (0.08)	1.1 (0.08)*	0.34 (0.04)	0.5 (0.06)
ePC(34:1)	7.7 (0.22)*	4.7 (0.10)	4.0 (0.19)	5.7 (0.24)*	4.0 (0.09)	4.5 (0.17)
ePC(34:2)	8.1 (0.19)*	5.3 (0.23)	5.1 (0.38)	9.1 (0.34)*	6.5 (0.22)	6.4 (0.39)
ePC(36:2)	14.6 (0.50)*	9.6 (0.29)	8.6 (0.63)	15.2 (0.39)*	11.0 (0.30)	10.4 (0.52)
ePC(36:3)	4.2 (0.11)*	2.7 (0.10)	2.5 (0.15)	4.1 (0.17)*	3.0 (0.11)	3.0 (0.17)
ePC(36:4)	12.9 (0.40)*	6.5 (0.21)	5.7 (0.31)	10.8 (0.31)*	5.8 (0.64)	6.0 (0.28)
ePC(38:2)	16.5 (0.76)*	7.7 (0.27)	6.0 (0.54)	14.3 (0.50)*	7.2 (0.38)	6.4 (0.55)
ePC(38:6)	12.0 (0.31)*	5.3 (0.13)	4.5 (0.24)	8.6 (0.24)*	4.5 (0.12)	5.0 (0.24)
ePC(40:4)	7.8 (0.30)*	4.4 (0.18)	4.1 (0.27)	8.3 (0.37)*	5.4 (0.21)	4.8 (0.25)
PC(38:6)	277.3 (7.95)*	225.5 (6.47)	188.3 (11.8)	194.2 (4.35)	185.1 (5.14)	174.8 (6.28)
PC(40:4)	11.0 (0.34)*	7.5 (0.23)	6.0 (0.37)**	11.7 (0.43)	6.6 (0.24)	5.4 (0.22)
PC(40:6)	98.3 (4.38)*	75.0 (1.98)	58.7 (3.39)**	82.4 (2.13)	63.7 (2.14)	66.1 (1.68)
PC(40:7)	407.8 (2.07)*	44.5 (1.29)	36.5 (1.93)	28.9 (1.54)	32.3 (1.27)	29.4 (0.76)
eLPC(16:0)	2.8 (0.10)*	1.6 (0.04)	1.5 (0.10)	2.2 (0.07)*	1.8 (0.03)	1.8 (0.06)
eLPC(18:0)	3.3 (0.11)*	2.0 (0.06)	1.8 (0.10)	3.1 (0.06)*	2.4 (0.03)	2.3 (0.07)
LPC(16:0)	208.8 (4.30)*	155.2 (1.38)	133.8 (6.59)	158.4 (4.25)	147.7 (3.02)	142.8 (3.34)
LPC(16:1)	5.4 (0.33)	5.8 (0.23)	4.6 (0.23)	2.6 (0.20)	3.3 (0.14)	4.4 (0.11)*
LPC(18:0)	113.4 (2.72)*	77.1 (1.00)	63.7 (3.18)	103.1 (3.37)*	76.6 (2.46)	80.6 (2.25)
LPC(18:1)	57.2 (2.68)*	47.7 (1.40)	39.3 (1.73)	32.6 (1.99)	32.1 (1.09)	32.7 (0.99)
LPC(18:2)	98.9 (2.53)*	84.03 (1.57)	81.7 (3.90)	103.2 (2.94)	90.8 (3.72)	95.2 (2.28)
LPC(20:5)	7.8 (0.25)	7.6 (0.19)	6.7 (0.35)	9.0 (0.55)*	8.0 (0.32)	7.1 (0.21)
LPC(20:4)	26.0 (1.40)*	31.2 (0.55)	33.5 (1.37)	33.1 (1.32)	31.3 (0.81)	42.1 (1.22) **
LPC(22:6)	16.0 (0.62)	17.1 (0.43)	17.0 (0.75)	17.3 (0.98)	16.7 (0.47)	20.3 (0.71) **
ePE(36:5)	3.9 (0.19)*	2.3 (0.10)	2.1 (0.10)	3.0 (0.13)*	2.3 (0.09)	2.1 (0.12)
ePE(40:6)	8.0 (0.41)*	3.9 (0.11)	3.8 (0.34)	5.5 (0.26)*	3.0 (0.13)	2.8 (0.14)
PE(36:2)	16.5 (0.96)*	4.9 (0.66)	3.6 (0.47)	8.9 (0.43)*	4.9 (0.37)	3.2 (0.48)
PE(36:4)	15.7 (0.70)*	8.6 (0.23)	7.6 (0.60)	8.3 (0.34)	7.9 (0.19)	6.6 (0.38)
PE(38:5)	11.3 (0.55)	7.4 (0.22)	7.9 (0.52)	5.4 (0.37)	6.0 (0.19)	5.7 (0.34)
PE(38:6)	20.8 (0.95)*	11.5 (0.25)	10.0 (0.83)	10.3 (0.44)	10.1 (0.33)	10.0 (0.73)
PE(40:6)	4.6 (0.43)*	2.0 (0.18)	1.7 (0.18)	2.4 (0.17)	2.2 (0.17)	2.3 (0.16)
PI(36:0)	0.18 (0.01)*	0.32 (0.00)	0.02 (0.00)	0.09 (0.00)*	0.02 (0.00)	0.01 (0.00)
PI(36:1)	4.34 (0.14)*	0.8 (0.06)	0.5 (0.10)**	2.1 (0.14)*	0.52 (0.07)	0.27 (0.03)
PI(36:2)	36.4 (1.04)*	7.3 (0.61)	4.3 (0.92)**	17.8 (0.96)*	4.8 (0.67)	2.5 (0.25)
PI(36:3)	17.7 (0.61)*	6.7 (0.44)	3.6 (0.55)**	7.9 (0.37)*	3.4 (0.36)	2.2 (0.22)
PI(38:5)	15.1 (0.72)*	10.1 (0.49)	5.8 (0.59)**	7.0 (0.42)*	4.4 (0.38)	4.1 (0.36)

Note: Species which were modulated in human samples are also examined in APOE-TR and EFAD mice. * indicates $p < 0.05$ for higher E2 vs. E3 and E4 for APOE-TR and for higher E2FAD vs. E3 and E4FAD. ** indicates $p < 0.05$ for higher E4 vs. E2 and E3 for APOE-TR and for E4FAD vs. E2FAD and E3FAD. These individual species are those identified in the human cohort.
